# Horizontal versus vertical charge and energy transfer in hybrid assemblies of semiconductor nanoparticles

**DOI:** 10.3762/bjnano.3.72

**Published:** 2012-09-06

**Authors:** Gilad Gotesman, Rahamim Guliamov, Ron Naaman

**Affiliations:** 1 Department of Chemical Physics, The Weizmann Institute of Science, Rehovot 76100, Israel

**Keywords:** charge transfer, energy transfer, nanoparticles, organic linker, quantum dots

## Abstract

We studied the photoluminescence and time-resolved photoluminescence from self-assembled bilayers of donor and acceptor nanoparticles (NPs) adsorbed on a quartz substrate through organic linkers. Charge and energy transfer processes within the assemblies were investigated as a function of the length of the dithiolated linker (DT) between the donors and acceptors. We found an unusual linker-length-dependency in the emission of the donors. This dependency may be explained by charge and energy transfer processes in the vertical direction (from the donors to the acceptors) that depend strongly on charge transfer processes occurring in the horizontal plane (within the monolayer of the acceptor), namely, parallel to the substrate.

## Introduction

Self-assembled structures of semiconductor nanoparticles (NPs) are viewed as a possible avenue for producing photovoltaic devices with efficient collection of light and charge separation processes [1]. Such structures can be produced by self-assembly of the NPs in a multilayer arrangement, by applying organic molecules as linkers. This concept enables high coverage and uniformity of the NP monolayers [2–3]. In all these types of devices, their performance is critically dependent on the ability to transfer charge and energy in the vertical direction, namely, normal to the substrate. Here we present evidence that charge and energy transfer processes in the vertical direction may strongly depend on charge transfer processes occurring in the horizontal plane, namely, parallel to the substrate. As a result, surprising distance-dependent transfer rates from the donors to the acceptors were observed, in which the transfer rate increases with the donor–acceptor distance.

The electron-transfer process in such hybrid organic–inorganic devices depends of course on the NPs, but also on the linker molecules [4]. Recently, many studies involving charge transfer through semiconductor NPs have been performed. Photoinduced charge transfer from NPs to organic dyes [5–6] and to conductive polymers [7–8] has been reported. Light-detectors and solar cells, based on NPs, employing carbon nanotubes [9], GaAs [10], or TiO_2_ devices [11–12] as the conductive substrates, have also been reported. Long-range resonance energy transfer was shown in mixtures of donor and acceptor close-packed arrays of NPs (quantum dot solids, QDS) [13–15]. Furthermore, studies on multilayered NP arrays, prepared by layer-by-layer electrostatic assembly (LBL) or Langmuir–Blodgett (LB) techniques, showed that the NPs funnel the absorbed energy from larger bandgap NPs to smaller ones [16–21]. Buhbut et al. even used NPs as nano-antennas in dye-sensitized solar cells. The NPs transfer the absorbed light (energy transfer) to the organic dye and thereby increase the absorption probability of the device [22]. Each transfer mechanism, charge or energy transfer, has a typical rate dependency on the distance between the donors and the acceptors. Therefore, distance-dependent studies enable one to reveal the type of transfer process. Wu et al. studied charge separation in type-II heterojunction assemblies of CdSe–CdTe NPs as a function of linker length [23]. They found that for longer linkers, the charge transfer efficiency is smaller. In another study, photogenerated exciton dissociation in PbS NP thin films was investigated when the NPs were linked by different linkers [24]. The linker length and the transfer rate were correlated, indicating tunneling charge transfer mechanisms at short distances and dipole–dipole energy transfer mechanisms at longer distances. In other studies, photoluminescence (PL) and PL-lifetime measurements of donor–acceptor NP bilayers, separated by polyelectrolyte layers, revealed that the energy transfer rate is inversely related to the distance. This is consistent with Förster’s theory for the dipole–dipole energy transfer mechanism in the donor–acceptor bilayers [25–26].

**Figure 1:**
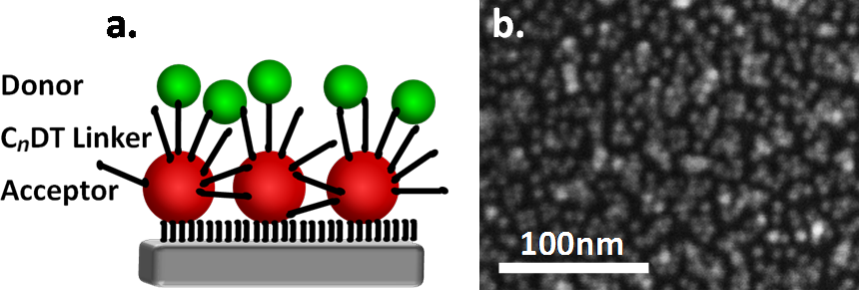
(a) A scheme of the investigated systems in which large CdSe NPs (acceptors) were assembled on quartz substrates by mercaptosilane monolayers. Dithiols of various lengths (C*_n_*DT) were then adsorbed on the first layer of the NPs followed by adsorption of a second layer of small CdSe NPs (donors). (b) SEM image of an acceptor NPs monolayer adsorbed on a Si/SiO_2_ substrate.

In the present study, we applied PL and PL-lifetime measurements to investigate charge and energy transfer processes in hybrid self-assembled bilayer structures of donor and acceptor semiconductor NPs bound through organic linkers (Figure 1a). This system is different from the QDS, LB and LBL systems, which were studied previously, since when the linkers are adsorbed, they may attach and couple neighboring NPs both horizontally and vertically. Other experiments, which were previously performed on similar assemblies of self-assembled NPs bilayers, were carried out with type-I core-shell NPs. In these NPs, the charges are localized in the core of the NP, due to the shell barrier, and only energy transfer processes are possible within the assemblies [20,27]. By using core-only NPs and linkers of different lengths, we found that the linkers strongly couple the acceptor NPs among themselves and enable charge transfer processes within the first layer. This coupling is dependent on the linker length and it may affect dramatically the energy transfer processes in the bilayer system containing donors and acceptors. We suggest a model that provides a possible explanation for the observed results.

## Experimental

The NP assemblies were prepared as described previously [4]. Briefly, a monolayer of (3-mercaptopropyl)trimethoxysilane was self-assembled on quartz (Qz, fused silica) substrates. Next, a monolayer of core-only CdSe NPs (~6.3 nm diameter, emission peak at 641 nm) was attached to the thiol tail group of the assembled molecules. The samples were then dipped into solutions of dithiolated alkyl molecules (C*_n_*DT) of various lengths. The organic molecules serve as linkers to which a second layer of NPs is attached. The linkers used were 1,3-propanedithiol (C_3_DT), 1,6-hexanedithiol (C_6_DT), 1,8-octanedithiol (C_8_DT), and 1,10-decanedithiol (C_10_DT). Control experiments were performed with molecules of the same lengths but with only one thiol group. These molecules will be denoted as monothiols (MT, e.g., C_6_MT for 1-hexanethiol). To form the second layer of NPs, the samples were immersed in a solution of smaller CdSe NPs (≈3.5 nm diameter, emission peak at 580 nm). Since charge and energy transfer processes are expected to occur from the small NPs (larger band gap) to the large ones (smaller band gap), we denoted the small and large NPs as donors and acceptors, respectively. Very dense monolayers of acceptor NPs were formed on the Qz substrate (see the SEM images in Figure 1b and Figure S3 in the Supporting Information File 1). However, the NPs are not evenly distributed over the entire surface; instead they form small clusters of about 10 NPs per cluster. Within each cluster the NPs are packed together. For more detailed experimental procedures please see Supporting Information File 1.

## Results and Discussion

**Figure 2:**
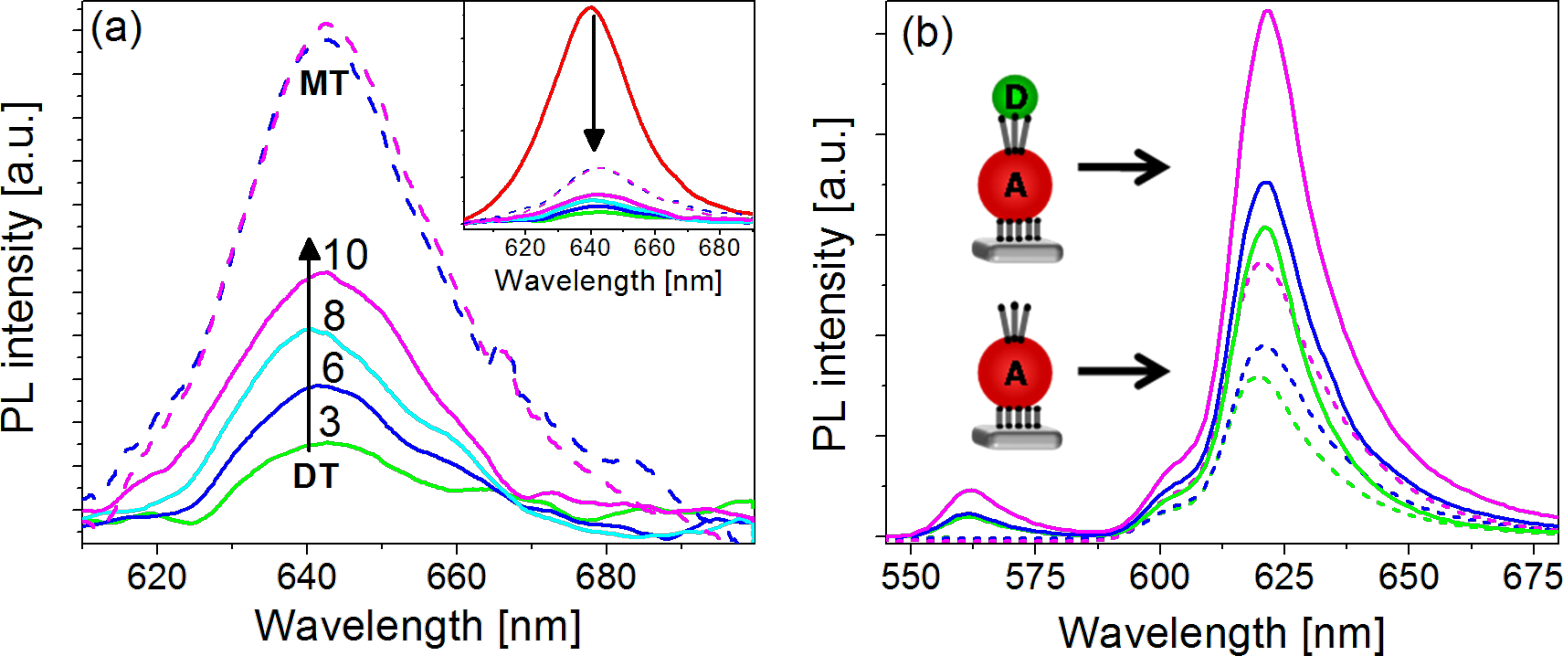
(a) PL spectra of acceptor NP monolayers at 300 K when covered with MT (dashed) or DT (solid) molecules of various lengths (C_3_-green, C_6_-blue, C_8_-cyan, C_10_-magenta). The inset shows the quenching of the untreated monolayer (red) upon the adsorption of the thiols. (b) PL spectra of acceptor NPs only (dotted) at 15 K covered with DT molecules of various lengths (C_3_DT-green, C_6_DT-blue, and C_10_DT-magenta) and spectra of donor–acceptor NP bilayers (solid) with the same linker molecules.

The effect of the adsorption of thiolated molecules (MT and DT) of various lengths on the PL intensity of the NP monolayers bound directly to the surface (referred to as the “acceptor” layer) is shown in Figure 2a. The PL measurements were performed at room temperature. The inset of Figure 2a reveals an efficient PL quenching, resulting from the adsorption of the thiolated molecules in comparison with an untreated sample. MT linkers were found to quench the PL signal by about 75%, and the quenching was independent of the linker length. This effect is well known for CdSe NPs; it results from the formation of surface traps owing to the thiol binding [28–31]. The PL quenching, which is independent of the MT length, indicates that the number of traps is the same for all linkers, and hence, the number of adsorbed molecules per particle is independent of the length of the linker. The adsorption of DT linkers resulted in more efficient quenching of the PL and was found to inversely correlate with the length of the linker: the shorter the linker, the larger the quenching effect. For example, quenching of approximately 95% was observed for the C_3_DT linker, whereas the quenching due to C_10_DT was about 85%. PL-lifetime measurements, which were performed at 15 K, revealed shortening in the lifetime of the acceptors upon adsorption of the various DT linkers.

We propose that the observed linker-dependent quenching for DT arises from cross-linking of the NPs within the layer and thereby facilitates charge transfer within the adsorbed monolayers of acceptor NPs in the horizontal plane. The energy transfer process, within the layer of the large NPs, probably takes place due to close packing of the NPs in the assembly process. This effect can be appreciated by the narrowing of the PL peak when comparing emission of the NPs in solution and in monolayer (Figure S5a, Supporting Information File 1). However, energy transfer alone cannot explain the observed linker-length-dependent quenching, since the energy transfer process depends on the distance between the NPs but not on the linker length (based on SEM images before and after the linker adsorption we assume that the NPs are fixed to the substrate and cannot move). It is also expected that with energy transfer, the PL will be red-shifted, and this shift, following the linker adsorption, has not been observed (Figure S5b, Supporting Information File 1). The charge transfer process (electron–hole separation) across the NP layers may occur because of slight differences in the NP sizes, which result in variations in the energy band gaps. This is a tunneling-based effect; therefore, it strongly depends on the length of the linker (as seen in Figure 2a). Dithiolated-linker length-dependent PL and PL-lifetime quenching, owing to charge separation within the NPs layer, was reported previously for PbS NP thin films [24].

To validate this effect, we studied NP monolayers having different coverage; the monolayers were covered with MT or DT linkers (Figure S4, Supporting Information File 1). Whereas for the highest coverage the NPs are almost in contact, for the lowest coverage the NPs are more separated, and the probability of adjacent NPs being linked within the layer decreases. Indeed, the PL results show that in the samples having the lowest coverage there is a minor difference between the effect of the MT and the DT, whereas in those with the highest coverage there is a large difference, as presented in Figure 2. These results confirm that an extra PL quenching mechanism is introduced by the adsorption of the DT linkers. This occurs most probably due to coupling between the NPs across the layer and the induction of charge transfer process between the NPs, when electrons are transferred from the relatively smaller NPs to those that are slightly larger.

For modeling the changes in PL from the DT-linked acceptor layer, we assume that *k*_AA_ (the charge separation rate in the acceptor layer, i.e., the layer bound directly to the substrate) is a function of the probability of finding a neighbor NP in the 2D layer, which increases with the area surrounding each particle, and of the tunneling-decay probability of charge transfer. Hence, the rate has the form:

[1]
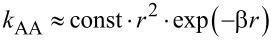


where *r* is the distance in angstrom and β is the effective tunneling decay coefficient in [angstrom]^−1^ (Supporting Information File 1). The PL from the acceptors should be inversely proportional to the charge separation rate in the acceptors layer.

[2]
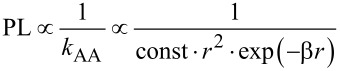


Hence, we fit the normalized maximum PL peak intensity of the DT-covered acceptor-NP monolayers as a function of the linker length (*r*) according to Equation 2. The fit is excellent (red dashed curve in Figure 4) and yields β of 0.42 [Å]^−1^. The value of the effective β is smaller than expected for alkyl chains (β ≈ 1 [Å]^−1^) and may indicate that the NPs are linked with several molecules in parallel. Since the PL signal is given in arbitrary units and since we are interested only in the ratio between signals, the absolute values of the other fitting parameters are meaningless. However, the fit provides two important insights. It indicates that the functional form of Equation 2 is consistent with the distance-dependent behavior and it provides the value for β.

The PL spectra, measured from various assemblies at 15 K, are shown in Figure 2b. The dotted curves are the PL spectra from the monolayers of the acceptors covered by DT linkers. The linker-length-dependent quenching trend is consistent with that measured at room temperature. When a layer of donor NPs is adsorbed on the layer of DT-covered acceptors, a new PL peak at the wavelength of the donors appears (≈560 nm) and the PL of the acceptor layer increases. The ratios of the area under the PL peak of the acceptors, with and without the donor layer, for the different DT linkers were equal. Furthermore, the intensity of the PL from the acceptors, when energy is transferred from the donors, follows the same linker dependency as observed for the acceptor layer alone.

It is well documented that the PL-lifetime of semiconductor NPs increases with decreasing temperature owing to less efficient electron–phonon coupling and to the emission from "dark states" [27,32–33]. In order to observe significant changes in the PL lifetime, as a result of charge and energy transfer, our measurements were performed at 15 K, with monitoring of the maximum of the PL peak. The data (dotted curves) was analyzed by a triple-exponential decay model, and by average lifetimes, which is a common practice [22–23,27,34]. This allows qualitative comparison of PL-decay profiles of the different assemblies. The fitting follows Equation 3 (solid line)

[3]
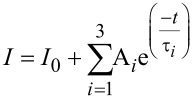


where τ*_i_* are the decay constants and A*_i_* are the pre-exponential parameters. The average lifetime for each sample (<τ>) was calculated by Equation 4.

[4]
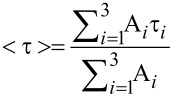


**Figure 3:**
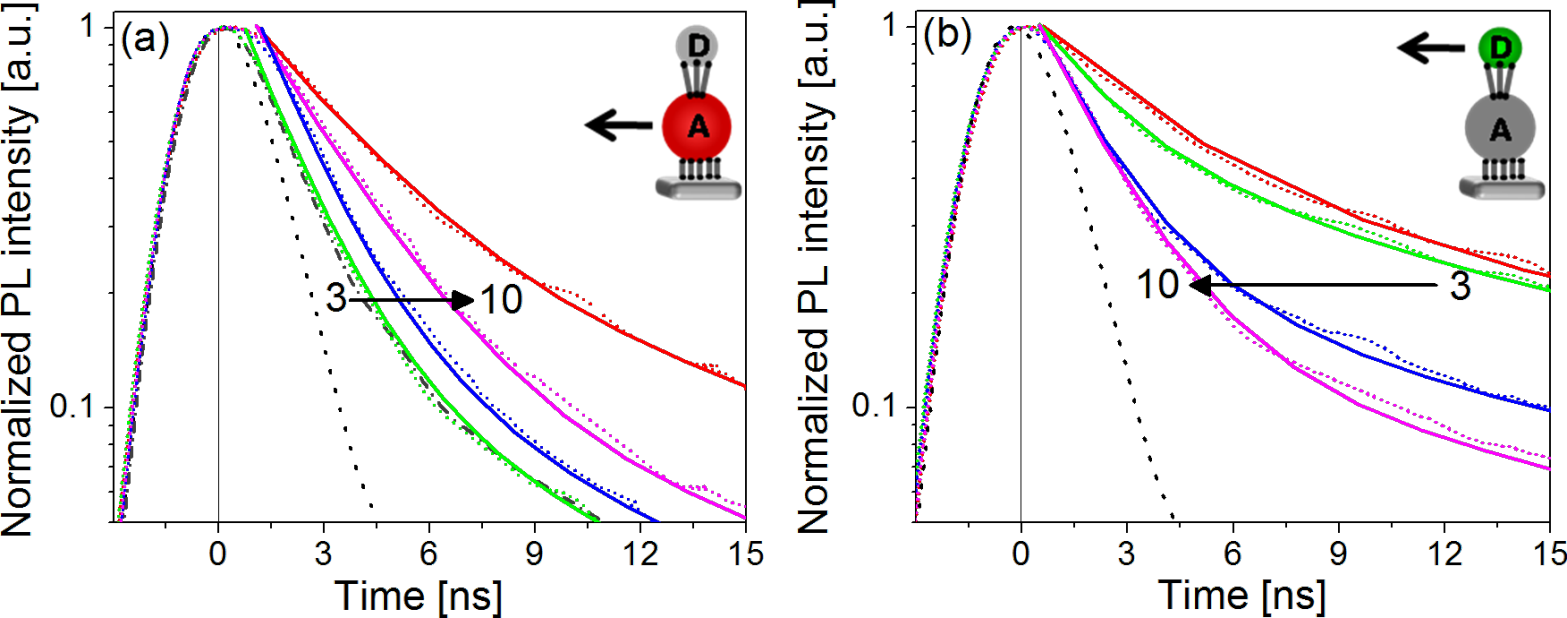
PL-lifetime measurements at 15 K of acceptor (a) and donor (b) NPs in the bilayer assemblies with various DT linkers (C_3_DT-green, C_6_DT-blue, and C_10_DT-magenta). The red curves represent control experiments of the PL-lifetime of a single NP monolayer of acceptors or donors, respectively. The lifetime of the acceptors coated with all types of DT is the same for all the linkers and is presented as a gray (dot–dash) curve in (a). We do not observe any change in the lifetime of the DT coated acceptor, since it is beyond the time resolution of the measuring setup. The dotted curves are the raw data and the solid ones are the three exponential fittings. The system response at the laser wavelength (460 nm) is represented by the dotted black curve. The intensities were normalized.

Figure 3 presents the PL-lifetime from the acceptor and the donor NPs in the bilayer samples as compared with a monolayer of acceptor or donor NPs only. The adsorption of the donor NP layer hardly changed the PL-lifetime of the acceptors for the bilayers with C_3_DT linkers (Figure 3a). However, for longer linkers, lengthening of the PL-lifetime was observed, which indicates that energy transfer processes took place. Based on absorption spectra and SEM images of the different samples (Supporting Information File 1), we concluded that the amount of donor NPs is almost equal in all samples and therefore the observed effects are not due to variations in the concentrations of the NPs. From Figure 3b it is evident that the presence of the layer of acceptors shortens the lifetime of the donors. However, surprisingly, the longer the linker, the shorter is the lifetime. Certainly, the observed linker-length dependency is counterintuitive.

The theoretical distance (*r*)-dependent transfer rates for energy and charge transfer, in donor–acceptor systems, are well known [19,35–38]. The energy transfer rate, *k*_ET_, in the case of the Förster mechanism (also referred to as FRET-dipole–dipole interaction), is known to follow the relation 
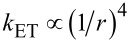
 for layered systems, and the charge transfer rate decays exponentially with the distance, for short distances up to about a few angstroms. When both electron and hole are being transferred simultaneously, the process is regarded as energy transfer of an exciton by the Dexter mechanism [39]. Hence, the Dexter transfer rate is proportional to the simultaneous tunneling of electrons and holes. The main differences between the Förster and Dexter mechanisms is that the Dexter mechanism demands an orbital overlap, between the donor and acceptor, besides a spectral overlap [7,40–41]. The second difference is that while FRET is a long-range transfer mechanism, the Dexter mechanism efficiency decays exponentially as a function of distance, and it is a short-range mechanism.

The donor–acceptor transfer rates in the bilayer assemblies were calculated, as in previous works, by using Equation 5 for each DT linker [27,42].

[5]
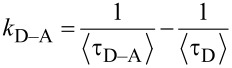


where <τ_D–A_> and <τ_D_> are the average lifetimes of the donors in the bilayer with a layer of acceptors and a monolayer with no acceptors, respectively.

**Figure 4:**
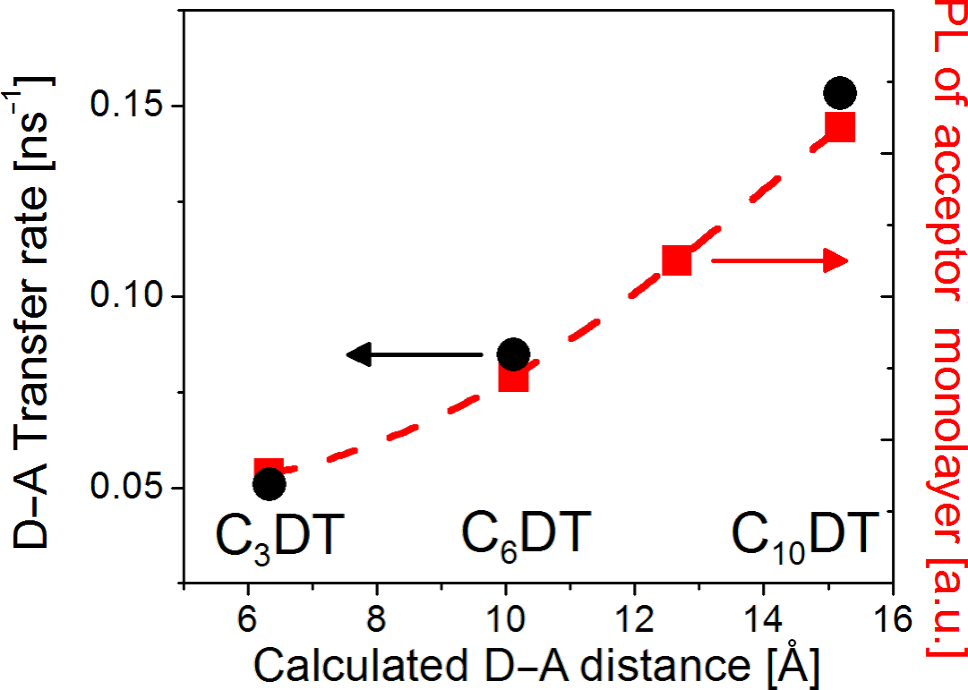
Energy transfer rates in the donor–acceptor NP bilayer (black) and normalized PL intensity from monolayers of acceptor NPs covered with various DT linkers (red) as a function of the linker length. The dashed red curve is the fit of the acceptor PL data to Equation 2 (β = 0.42 Å^−1^, with a coefficient of determination of: R^2^ = 0.99991 and a confidence level of parameters of 95%).

The energy transfer rate constants between the donors and acceptors (D–A) versus their distances are presented in Figure 4 and are compared with the normalized PL intensity of the acceptors alone in the monolayer, following the adsorption of the dithiols. We assume that the distance between the donor and acceptor NPs is the same as the theoretical length of the linkers (for the distance calculation, see Supporting Information File 1). The results indicate that the donor–acceptor transfer rate increases with the length of the DT linking molecules (i.e., the donor–acceptor distance). This contradicts the expected distance dependency. It was previously shown that the concentration affects the energy transfer efficiency in NP bilayers only below an acceptor/donor concentration ratio of 1:1. Above this ratio, the energy transfer efficiency reaches a maximum and hardly depends on the concentration [26,43]. Our assemblies have an acceptor/donor concentration ratio above one (since the adsorption efficiency of the second layer is lower) and therefore they are not expected to exhibit concentration effects. In addition, absorption spectra and SEM results indicate that the concentration ratio is about the same for all bilayers (Supporting Information File 1).

Another possible explanation for the observed trend is that the number of thiols that bind to each donor NP depends on the length of the molecule. To test this idea, we conducted a control experiment in which we used donor NPs of the same size as before but with a different capping ligand. The ligand in this case was tetradecyl phosphonic acid (TDPA), which binds more strongly to the NP surface [44]. We assumed that if the number of linkers that bind to the top NP induces an effect, it will be influenced by the probability to bind to these NPs. Here, with the TDPA capping on the donor NPs, it is much harder to replace the original capping by a thiol bond [44]. Therefore, a different length dependence of the quenching process is expected. However, the PL-lifetime trend from these assemblies was similar to the one presented in Figure 3 (Supporting Information File 1). It is important to realize that in the two cases the linker-length-dependent trend in lifetimes is the same, although the values are somewhat different, probably due to differences in the particles concentration. Therefore, we conclude that the observed effect is not due to a different number of thiols bound to each donor.

Hence, there must be another rate-determining parameter besides distance, concentration, or the number of binding groups, which controls the vertical transfer processes. It is also evident from Figure 4 that the trend regarding the change in the transfer rate, as a function of the linker length, follows the trend of the normalized PL intensity dependence on the DT length, in the DT-covered acceptor monolayer. As was indicated above, this linker-dependent quenching is probably caused by coupling of the acceptor NPs among themselves through the DT linkers, enabling horizontal charge transfer within the layer.

**Scheme 1:**
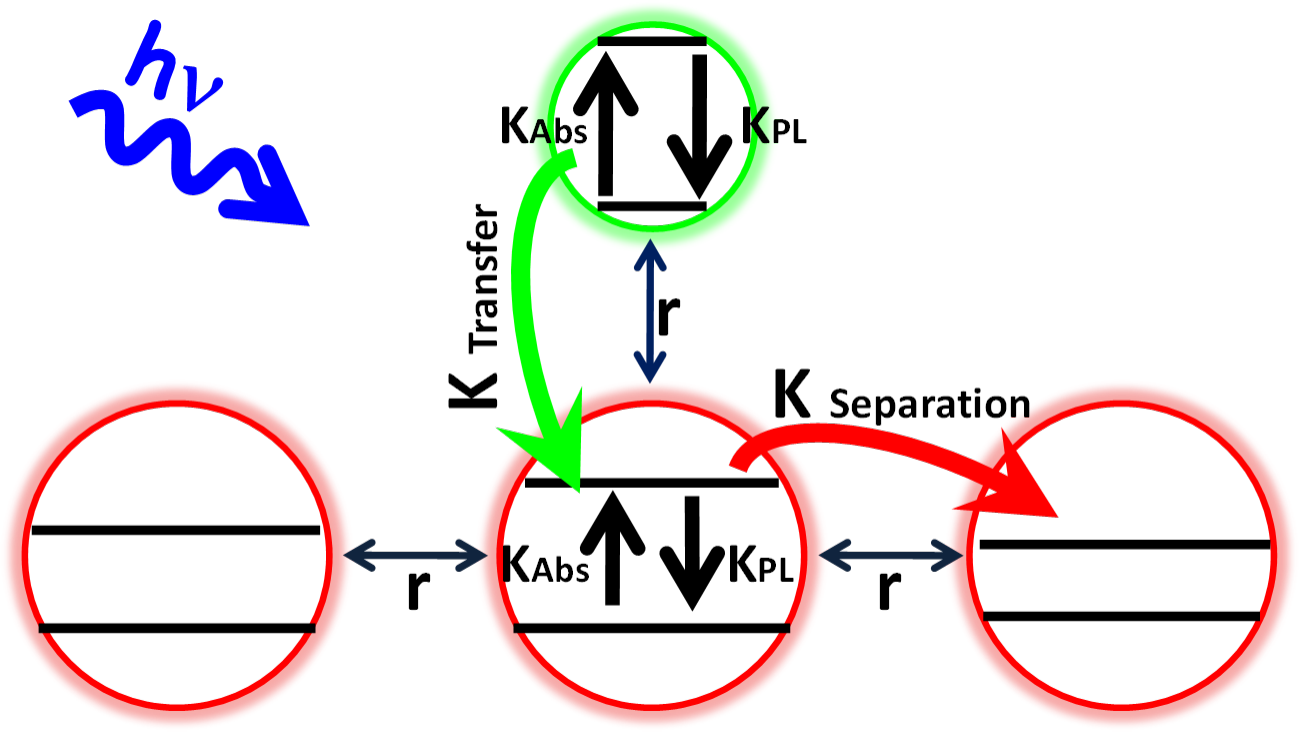
NPs Bilayer composed of donor NPs (green) adsorbed on acceptor NPs (red) through DT linkers of length (*r*). The linkers connect the NPs at the top layer to the bottom layer, as well as connecting the NPs within the bottom layer itself. Absorption and PL rates (*k*_Abs_, *k*_PL_, respectively) are defined for each NP layer, alongside the horizontal charge separation rate in the acceptor layer (*k*_A–A_) and the vertical donor-acceptor transfer rate (*k*_D–A_).

Scheme 1 depicts a model that rationalizes the unusual observations. This model is based on the assumption that the acceptors are coupled to each other by the DT linkers. This coupling allows charge transfer across the layer and is stronger for the shorter DT. This charge transfer process results in part of the acceptors being charged, until charge recombination occurs. As long as these NPs are charged, the charge or exciton transfer (in the Dexter mechanism the electron and hole are transferred simultaneously) is less efficient to them due to the Columbic repulsion, which results in a reduction in the overlap of the wave functions of the donor and the charged NP. However, the transiently charged NPs can accept energy in the Förster mechanism (dipole–dipole interaction). It is important to realize that due to the charged acceptor, the barrier for hole transfer increases, and as a result the entire exciton transfer becomes less efficient. Hence, the rate of the charge transfer, between the donors and the acceptors, depends on the number of acceptor NPs that remain in their ground state uncharged. This number of ground-state acceptors depends inversely on the coupling efficiency of the DT linkers, which decreases with an increase of the linker length. Hence, the donor–acceptor energy transfer rates increase with the donor–acceptor distance.

The fact that the donor–acceptor transfer rate (*k*_DA_) follows exactly the acceptors PL quenching trend (Figure 4) implies the relation:

[6]
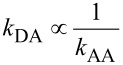


Therefore we conclude that these two processes are competing and that the charge separation process is a rate-determining step for the donor–acceptor transfer process.

## Conclusion

In summary, distance-dependent charge and energy transfer studies in self-assembled bilayers of acceptors and donors of CdSe NPs, linked through DT molecules, were performed by PL and PL-lifetime techniques. Importantly, we found that the PL of the acceptor NPs layer is quenched following the adsorption of the DT linkers and that the quenching depends on the linker length. The shorter the linker, the more efficient is the quenching. This quenching is caused, most probably, by charge transfer among the acceptor NPs in the layer (horizontally) mediated by the DT linkers. The donor–acceptor energy transfer rates in the bilayer samples (vertically) increased when the linker length increased. This trend is opposite to that predicted theoretically for distance-dependent charge or energy transfer. In considering the observed trends, we present a model that suggests that the donor–acceptor transfer rate can be explained as being controlled by the ability of the acceptors to accept energy from the donors. Namely, only acceptors in the ground state are able to receive excitons from the donors. Therefore, this ability is controlled by the interactions among the acceptor NPs that are coupled through the DT molecules. The observed results indicate that here the energy transfer process probably occurs by the Dexter mechanism, since the Förster mechanism alone cannot explain the observed trends. The surprising findings presented here are very important for designing future self-assembled NP devices. We demonstrated that in addition to their structural role as assembly agents, the linker molecules play a key role in defining the photophysical properties of the system.

## Supporting Information

File 1 Experimental procedures, UV–vis spectra, SEM images, PL-lifetime data, and the model development equations.
