# Supplementary material for: Horizontal versus vertical charge and energy transfer in hybrid assemblies of semiconductor nanoparticles
Source: Beilstein J Nanotechnol. 2012 Sep 6;3:629–36. doi: 10.3762/bjnano.3.72 (PMC3458609; doi:10.3762/bjnano.3.72)
Supplement: File 1 — Experimental procedures, UV–vis spectra, SEM images, PL-lifetime data, and the model development equations. [file Beilstein_J_Nanotechnol-03-629-s001.pdf]

Supporting Information

for

# Horizontal versus vertical charge and energy transfer in hybrid assemblies of semiconductor nanoparticles

Gilad Gotesman, Rahamim Guliamov and Ron Naaman*

Address: Department of Chemical Physics, The Weizmann Institute of Science, Rehovot 76100, Israel

Email: Ron Naaman - ron.naaman@weizmann.ac.il

* Corresponding author

**Experimental procedures, UV–vis spectra, SEM images, PL-lifetime data, and the model development equations.**

**Experimental:**

*Synthesis of nanoparticles:* The synthesis of CdSe nanocrystals was performed by following the noncoordinating solvent procedure. The whole synthesis was carried out using the Schlenk technique. Briefly, a mixture of cadmium oxide (0.0128 g, 0.1 mM), hexadecylamine (HDA, 1.3 mL), oleic acid (0.3 mL), and technical grade 1-octadecene (ODE-4 mL) was dried and degassed under vacuum at 120 °C for 30 min in a 50 mL three-neck flask. The solution was heated under argon to 280 °C, and then the stock solution of trioctylphosphine (TOP)-selenium, prepared by dissolving elemental selenium (0.078 g, 0.1 mM) in 2 mL TOP, was quickly injected into the hot solution under vigorous stirring. The growth temperature was then reduced to 250 °C until the dots reached the desired diameter (about 20 min). Typically, this reaction generates CdSe nanocrystals of about 3.5 nm in size, with the first absorption peak around 570 nm. For larger nanocrystals, the 3.5 nm particles were further grown using a layer-by-layer growth technique in a one-pot synthesis. The injection solutions used for the CdSe growth were 0.1 M of cadmium oleate and 0.1 M of selenium in TOP. For each injection, a calculated amount of a given injection solution was taken and injected in a dropwise manner into the solution containing synthesized CdSe nanocrystals. Three layers of CdSe were grown at 250–260 °C and annealed at 270 °C for 20 min. Following the annealing, the solution was cooled to room temperature and stored in a freezer until the precipitation procedure. Separation of unreacted precursors from the nanocrystals was performed by repeated precipitation (twice in most cases) and redispersed with acetone/methanol and toluene, respectively. Finally, the particles were stored in toluene.

*Self-assembled monolayers on quartz:* Quartz slides (fused silica - Chemglass) were cleaned prior to silanization by boiling in acetone and ethanol for 10 min each, followed by UV/ozone oxidation (UVOCS) for 20 min. The samples were then soaked in ethanol for 15 min and dried with $N_2$ flow. Monolayers of (3-mercaptopropyl)trimethoxysilane (>97%, Fluka) were formed by dipping the samples into a 1 mM solution in bicyclohexyl (BCH, 99%, Aldrich) for 1 h followed by sonicating the sample in toluene for 1 min. This process of dipping and sonicating was repeated twice to ensure full monolayer coverage. Solvents were reagent grade or better and were purchased from Merck, Baker, or Bio-Lab.

*Monolayers of nanoparticles:* In order to form a monolayer from CdSe NPs, the SAM-coated substrates were immersed for 3 h in 0.5–10 μM core-only CdSe640 solutions in anhydrous toluene (99.8%, Aldrich). The samples were then sonicated twice (30 sec each) in toluene to remove excess NP solution and dried rapidly under $N_2$ flow.

*Linker molecule adsorption:* The samples with the NP monolayers were immersed in a 1 mM solution of dithiolated alkyl linker molecules in methanol for 3 h followed by rinsing with ethanol and drying under $N_2$ flow. For linkers we used the following molecules: 1,3-propanedithiol ($C_3$DT, 99% Aldrich), 1,6-hexanedithiol ($C_6$DT, 97% Alfa Aesar), 1,8-octanedithiol ($C_8$DT, 98% Alfa Aesar),1,10-decanedithiol ($C_{10}$DT, 99% Alfa Aesar). To form the second layer of nanoparticles, the samples were then immersed in a CdSe585 nanoparticle solution according to the procedure described above.

*Electron microscopy and NP coverage:* High-resolution scanning electron microscope (SEM) in-lens-detector imaging was carried out with a LEO-Supra 55 VP HRSEM. The images were analyzed to calculate the coverage density using ImageJ (a freeware program available at rsb.info.nih.gov/ij). High-magnification images with very uniform NP monolayers were used and 10 precise squares of 100 × 100 nm were drawn on each image using ImageJ software. The number of NPs in each square was counted manually and the average and standard deviations were calculated. The results were normalized to units of the number of particles per micrometer square.

*Photoluminescence measurements:* The PL measurements were performed at 300 K using a LabRam HR800-PL spectrofluorimeter microscope (Horiba Jobin–Yvon) with an excitation source at 457 nm (argon-ion CW laser ~15 mW/cm$^2$). The incident light impinged on the surface at an angle of about 70° relative to the sample surface and the PL spectra were collected by the microscope (with 5× high working distance lens). To maintain the stability of the signal, we used a microscope-Linkam stage, where the samples were sealed during the measurements under a constant flow of $N_2$.

*Photoluminescence life-time measurements:* The PL life-time measurements were performed using the experimental setup shown in Figure S1. Briefly, the samples were placed on the cold finger of a He-cryostat cooled by cryopump (Oxford Instruments). The system was equipped with a close-loop temperature-controlling system (Scientific Instruments), which maintained the required sample temperature with ±0.2 K accuracy in the range of 15–200 K. Sample excitation was performed using 7 ns laser pulses at 460 nm wavelength and a 10 Hz repetition rate (Spectra Physics MOPO-PO optical parametric oscillator pumped by Spectra Physics Quanta-Ray Pro-230 Nd:YAG laser). Photoluminescence was spectrally resolved using a spectrometer (Horiba iHR-320). The photoluminescence signal was measured using a fast photomultiplier tube (PMT, Hamamatsu), a fast wide-band preamplifier (Femto), and a wide-band oscilloscope (LeCroy). Photoluminescence from the sample was collected and focused on the spectrometer input slit using two lenses. Scope synchronization was performed using a separate amplified silicon photodiode connected to a scope triggering input; recorded and stored data were analyzed by a multi-exponent decay equation to yield the PL lifetime of the NPs.

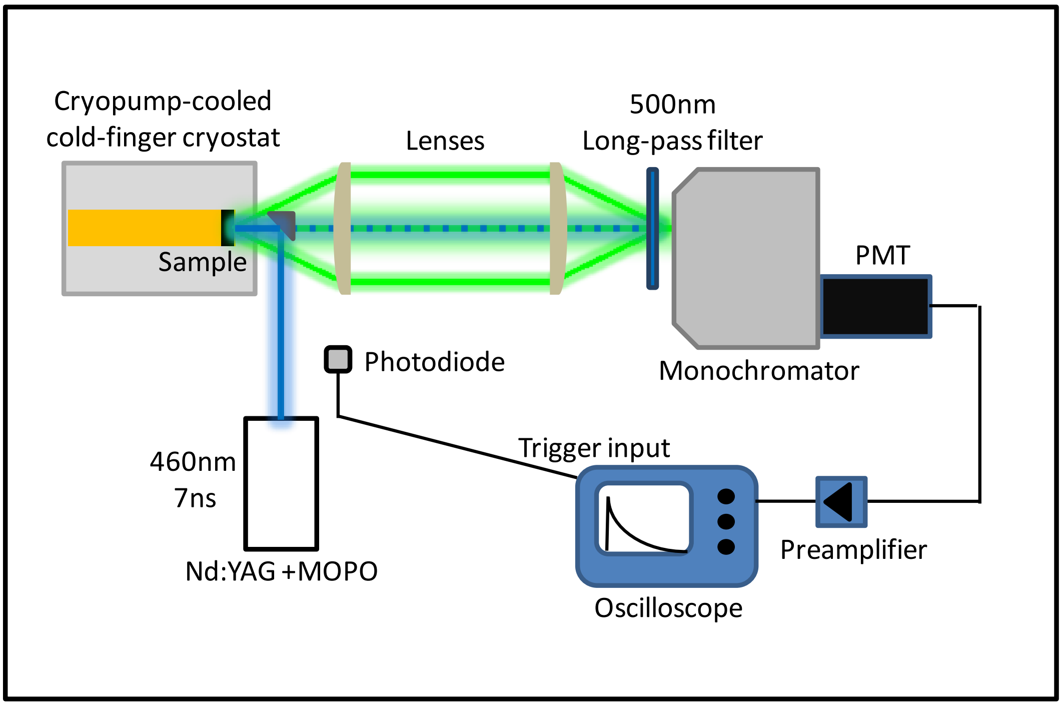


**Figure S1:** PL-lifetime experimental setup.

*Characterization*

The absorption and emission spectra of the NPs in solution are presented in Figure S2. The first absorption peak of the donor NPs (black) and their emissions are at 568 nm and 580 nm, respectively, whereas for the acceptor ones (red) the peaks are at 612 nm and 641 nm, respectively.

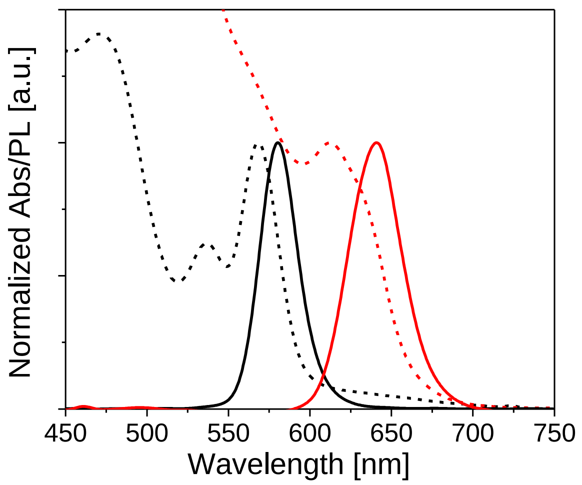


**Figure S2:** Absorption (dashed lines) and emission (solid lines) spectra of donor (black) CdSe NPs and acceptor (red) CdSe NP solutions.

This set of nanoparticles was chosen for several reasons. Both particles absorb blue light; thus, it is possible to excite both of them with a 457 nm laser. There is an overlap between the emission of the donor NPs and the absorption of the acceptor ones. This overlap allows energy transfer between the nanoparticles. Nevertheless, the emission peaks of the NPs are spectrally far enough from each other and it is possible to detect the PL of each NP layer separately.

The quality of the NP monolayers was characterized by high-resolution SEM. Figure S3 shows SEM images of acceptor NPs (CdSe641) at two magnifications. In order to prevent charging effects, the samples for the SEM imaging were performed on $Si/SiO_2$ substrates (highly doped, <100>, n-type, University Wafers, Boston, MA). It was noted that the CdSe NPs form a very dense monolayer on the silane-coated substrate. However, the NPs are not closely packed over the entire surface but instead form small islands (~10 NPs per island) of NP arrays. In these arrays, the NPs are adsorbed very close to each other. This closed packing of NPs in the monolayer enables the linker molecules to link between adjacent NPs. The adsorption of the thiolated linkers on the NPs layers does not change the structures of the arrays.

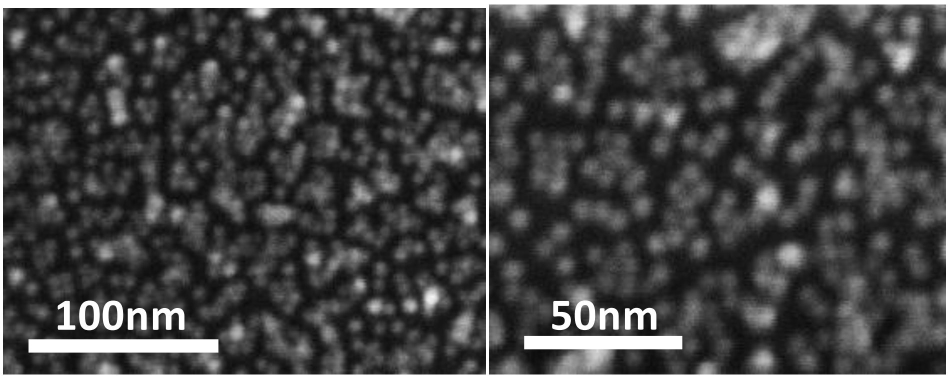


**Figure S3:** SEM images of acceptor (CdSe641) NP monolayers adsorbed on $Si/SiO_2$ substrate.

In order to prove the claim that the DT linkers form another quenching mechanism, besides the thiol trap states, we studied large CdSe NPs monolayers having different coverages. By varying the adsorption time of the NPs, we achieved three different assemblies with the following coverages: A. 2500 NP/$\mu m^2$, B. 5200 NP/$\mu m^2$, and C. 5900 NP/$\mu m^2$. SEM images of the different assemblies are presented in Figure S4a. The assemblies with high coverage (B, C) do not form a full and well-organized layer but instead form clusters of NPs, in which the NPs are positioned very close to each other (≈1 nm). In the sample with the lowest coverage (A), there are few clusters adsorbed on the surface, but there are many "single" isolated particles, and the distance between the NPs is larger. Therefore, the linking between the NPs in sample A should be minor and the effect should not be seen.

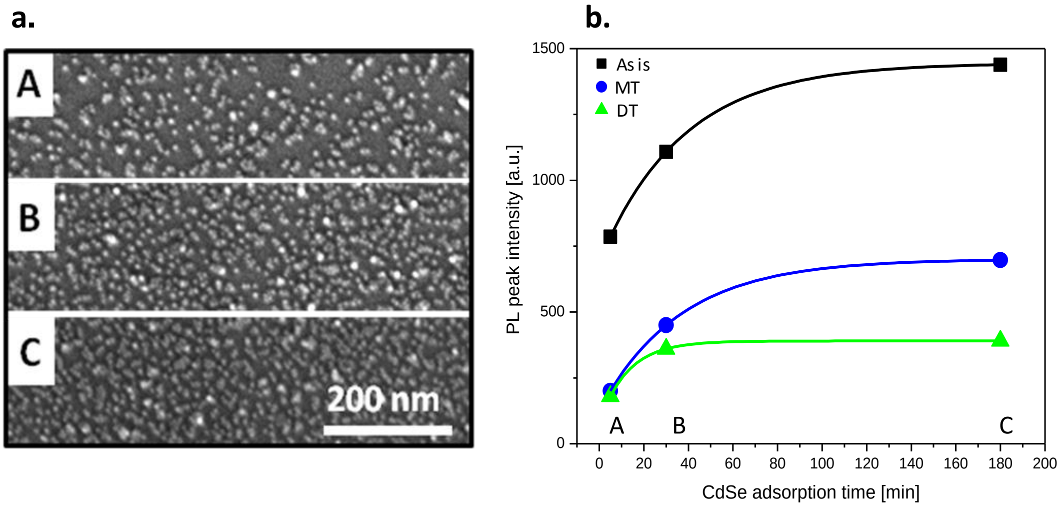


**Figure S4:** (a. left) Three SEM images of NP assemblies adsorbed for different times and with different coverages: A. 2500 NP/$\mu m^2$, B. 5200 NP/$\mu m^2$, C. 5900 NP/$\mu m^2$. (b. right) PL peak intensity for the three assemblies (A, B and C) when measured as is with TOPO ligands (black), exchanged with MT linker (blue), and exchanged with DT linker (green).

Figure S4b shows that the PL from the NP monolayer that is covered with the original ligands (black) increases when the NP adsorption time (NP coverage) is increased. When MT (blue) and DT (green), both having a length of ten carbons, are adsorbed on top of the NP layer, the PL signal decreases. In cases C and B, when the NPs form clusters on the surface, it was noted that the DT linker quenches the PL more efficiently than does the MT linker. But in case A, where the NPs are separated from each other, the PL from the two assemblies (MT and DT) is almost equal. This result confirms that an extra PL quenching mechanism is introduced by the adsorption of the DT linker, probably due to coupling between the nanoparticles across the layer, which increases the charge delocalization in the NP layer.

In order to determine whether an energy transfer process exists in the acceptor NP layer compared to the NPs in solution, the PL was normalized and shown in Figure S5a. Almost no red-shift is observed due to the adsorption on the surface; however, a narrowing of the PL peak is observed, which is an indication of an energy transfer process within the condensed NP layer. When adsorbing DT/MT linkers on the NPs, we observe a small red-shift for both linkers. The normalized data for the acceptor PL covered with the different DT linkers are presented in Figure S5b. Here, it is clear that there is no red shift of the PL peak due to the adsorption of the various DT linkers, within the error of the measurement. Therefore, we concluded that the adsorption of the DT linkers does not affect energy transfer processes within the acceptor NP layer.

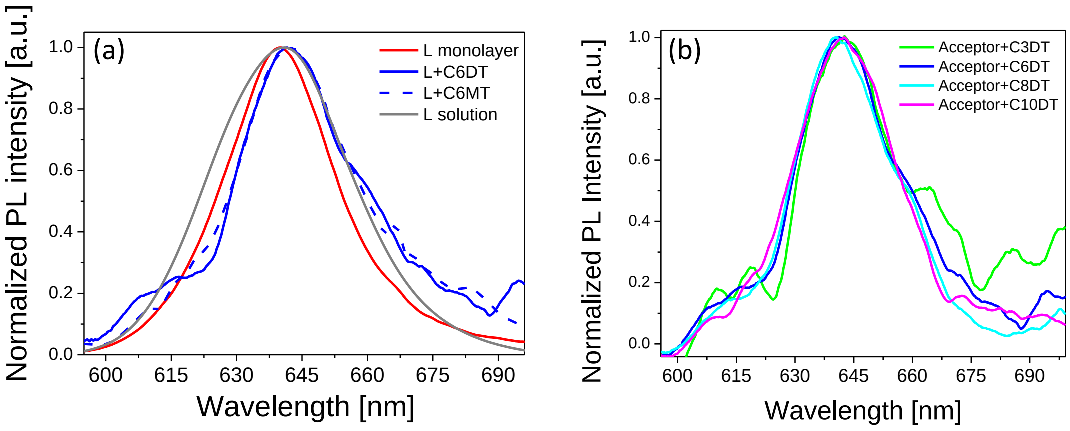


**Figure S5:** Normalized PL signal from a. NPs solution (gray), monolayer (red) and monolayer covered with $C_6$DT and $C_6$MT (solid and dashed blue respectively). b. An acceptor NP monolayer covered with various DT linkers of various lengths ($C_3$-green, $C_6$-blue, $C_8$-cyan, $C_{10}$-magenta).

The adsorption of the first NP monolayer was very reproducible. In order to check whether the number of NPs in the second layer was equal for all DT linker molecules, we imaged bilayer samples with different DT linkers. Since the larger NPs have a better SEM contrast than do the smaller ones (when placed at the second layer), we first adsorbed the donor NPs and then the acceptor ones through DT linkers. In order to increase the differences between the NPs, we used very small NPs (CdSe524, ≈2.5 nm) for the first layer. The SEM images in Figure S6 show the various assemblies with the different linkers. The brighter spots in the bilayer samples represent the larger top NPs. The bottom NPs cannot be seen since they are very small and they lose almost all their SEM contrast due to the DT adsorption. The coverage of the top NPs was calculated and is presented in Figure S7. It is clear that the number of top NPs in these systems is almost equal and that the adsorption efficiency through the various linkers is almost the same.

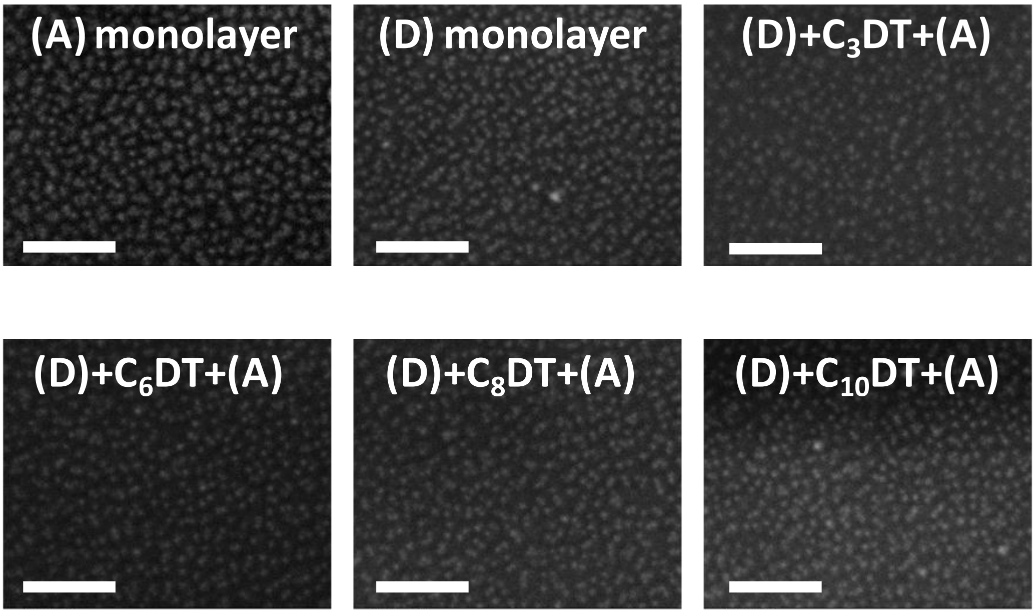


**Figure S6:** SEM images of acceptor (CdSe641) NPs, donor (CdSe524) NPs, and acceptors on a DT linker on donor bilayers with various linkers adsorbed on $Si/SiO_2$ substrate. The scale bar for all the images is 100 nm.

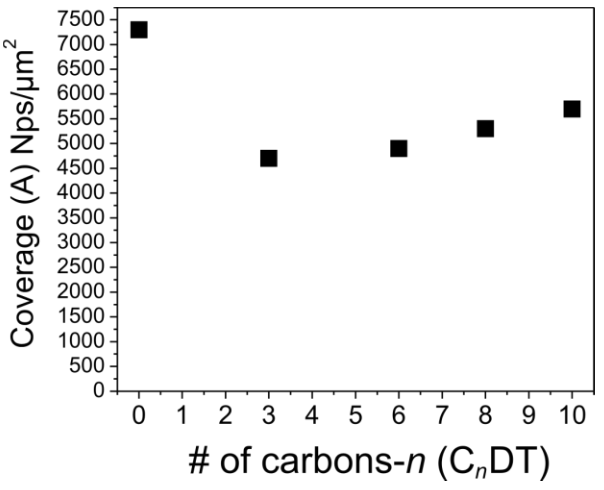


**Figure S7:** Coverage of acceptor (CdSe641) NPs in the monolayer ($n = 0$) and bilayers ($n$ = 3,6,8,10 carbons) of NPs for the samples presented in Figure S4.

The DT linker length was calculated by ChemDraw software and is presented in Table S1.

**Table S1:** calculated length and schemes of DT linkers.

| linker molecule | symbol | calculated thiol – thiol distance [Å] |
|---|---|---|
| 1,3-propyldithiol | $C_3DT$ | 5.5 |
| 1,6-hexanedithiol | $C_6DT$ | 9.32 |
| 1,8-octanedithiol | $C_8DT$ | 11.84 |
| 1,10-decanedithiol | $C_{10}DT$ | 14.29 |

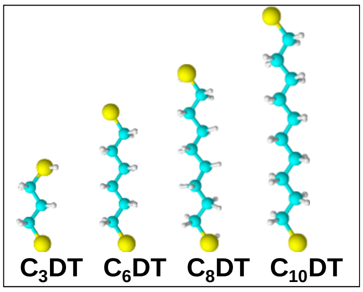


In order to prove that the number of top NP layers is equal for the various linkers, UV–vis absorption spectra for bilayers with C3DT and C10DT were taken. The results, which are presented in Figure S8, show that the absorption spectra of these assemblies overlap, which indicates that both samples have the same number of NPs.

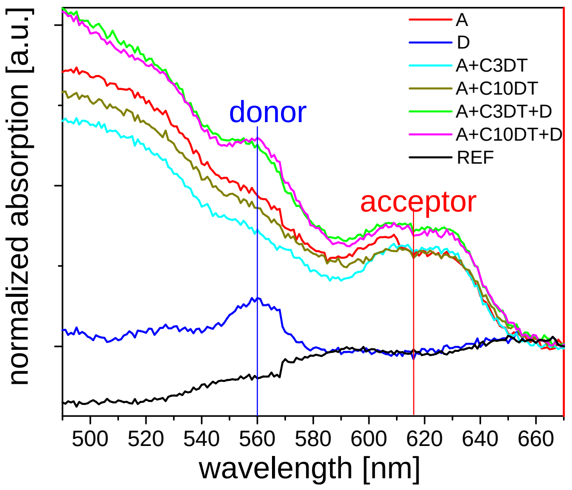


**Figure S8:** Absorption spectra of the various assemblies.

The PL-lifetime measurements were performed using a 460 nm laser with a 7 ns pulse. Since charge and energy transfer processes are very rapid at room temperature, the measurements were performed at 15 K in order to get the maximum difference between the samples. We performed the measurements up to 65 K and the same trends were observed at this temperature range (see Figure S9). The normalized data were fitted by triple-exponent functions and average lifetimes were calculated. A summary of the fitting parameters and average lifetimes is presented below in Table 2. The measurements were performed for long decay times (as seen in Figure S10a), but since most of the differences between the samples occurred during the first 15–30 ns, the presented data in the article are in this range (Figure S10b).

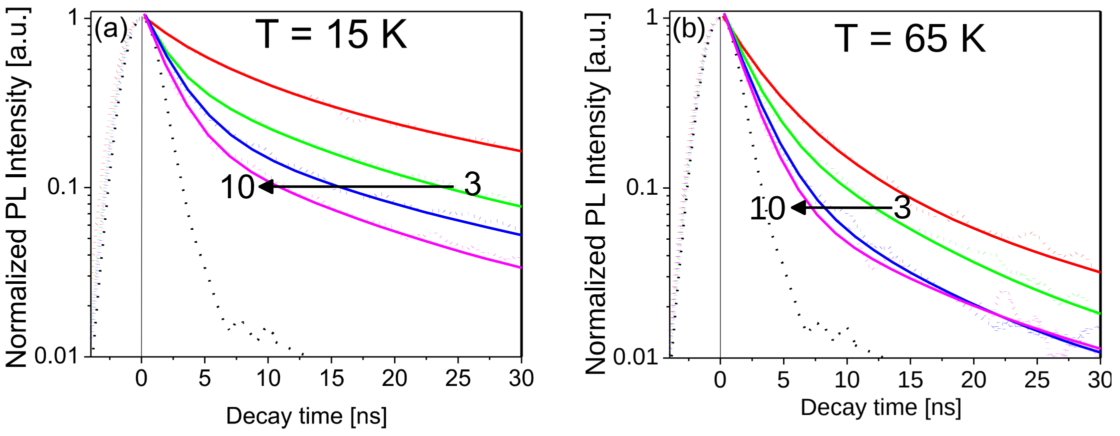


**Figure S9:** Normalized PL-lifetime measurements at 15 K (a) and at 65 K (b) of donor NPs in the bilayer assemblies using various DT linkers ($C_3$DT-green, $C_6$DT-blue, and $C_{10}$DT-magenta). The red curves represent control experiments of the PL-lifetime of a single NP monolayer of donors. The dotted curves are the raw data and the solid ones are the three exponential fittings. The system response at the laser wavelength (460 nm) is represented by the dotted black curve.

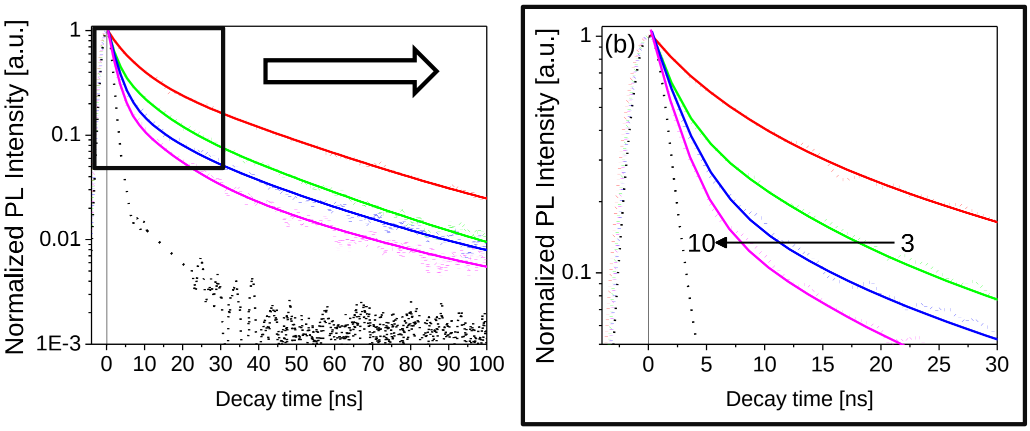


**Figure S10:** Normalized PL-lifetime measurements at 15 K of donor NPs in the bilayer assemblies using various DT linkers ($C_3$DT-green, $C_6$DT-blue, and $C_{10}$DT-magenta). The red curves represent control experiments of the PL-lifetime of a single NP monolayer of donors. The dotted curves are the raw data and the solid ones are the three exponential fittings. The system response at the laser wavelength (460 nm) is represented by the dotted black curve.

In order to rule out an effect of the amount of thiols binding to the donor top layer, we performed the same experiments with NPs having different capping ligands. We used tetradecyl phosphonic acid (TDPA) capped NPs since their ligands bind to their surface much more strongly. The observed trend (presented in Figure S11) is similar to the one observed with the HDA-capped NPs. Therefore, we concluded that the amount of thiol bonds in relation to the donor NPs cannot cause the observed trends.

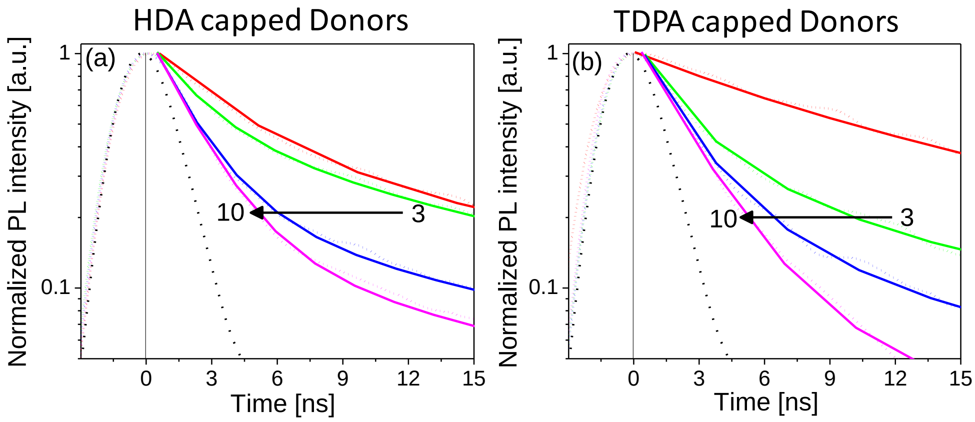


**Figure S11:** Normalized PL-lifetime measurements at 15 K of donor NPs with (a) HDA capping and (b) TDPA capping in the bilayer assemblies using various DT linkers ($C_3$DT-green, $C_6$DT-blue, and $C_{10}$DT-magenta). The red curves represent control experiments of the PL-lifetime of a single NP monolayer of donors with each capping molecule, respectively. The dotted curves are the normalized raw data and the solid ones are the three exponential fittings. The system response at the laser wavelength (460 nm) is represented by the dotted black curve.

All the results of the PL-lifetime fittings and the donor-acceptor transfer efficiency are presented in Table S2.

**Table S2:** Summary of the lifetime parameters of the fitted data by the triple-exponential function ($A$, $\tau$), the average lifetime ($\langle\tau\rangle$) of each sample, and the energy transfer efficiency ($E_{D\text{–}A}$) and rate ($k_{D\text{–}A}$) of the donor–acceptor system.

| | sample | A1 | τ1, [ns] | A1 | τ2, [ns] | A3 | τ3, [ns] | $\langle\tau\rangle$, [ns] | $E_{D\text{-}A}$ | $k_{D\text{-}A}$, [ns$^{-1}$] |
|---|---|---|---|---|---|---|---|---|---|---|
| PL from Acceptor **(A)** | **A** monolayer | 0.5 | 2.99 | 0.49 | 2.99 | 0.265 | 15.45 | 5.62 | | |
| | **A**+$C_3$DT+D | 1.13 | 1.66 | 0.14 | 6.82 | 0.023 | 41.56 | 2.933 | | |
| | **A**+$C_6$DT+D | 1.01 | 1.8 | 0.21 | 5.2 | 0.032 | 28.93 | 3.06 | / | / |
| | **A**+$C_{10}$DT+D | 1.07 | 1.67 | 0.17 | 5.73 | 0.038 | 38.45 | 3.31 | | |
| PL from Donor **(D)** | **D** monolayer | 0.61 | 2.08 | 0.32 | 7.63 | 0.23 | 35.44 | 10.22 | | |
| | A+$C_3$DT+**D** | 0.74 | 3.55 | 0.31 | 16.94 | 0.001 | 54.05 | 7.54 | 0.262 | 0.034 |
| | A+$C_6$DT+**D** | 0.94 | 1.88 | 0.165 | 6.4 | 0.12 | 33.38 | 5.62 | 0.45 | 0.08 |
| | A+$C_{10}$DT+**D** | 1.15 | 2.45 | 0.073 | 7.85 | 0.043 | 30.51 | 3.716 | 0.636 | 0.171 |

## PL quenching in the acceptor layer

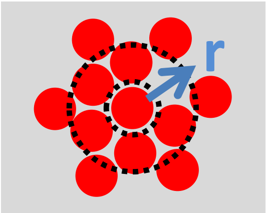


Assuming that the PL from the acceptors is inversely proportional to the separation rate $k_{AA}$.

We now assume that:

$$k_{AA} = P_n(r)\cdot T(r)$$

where $P_n(r)$ is the zero-order probability to find an acceptor NP at a distance ($r$) from a certain point and $T(r)$ is the probability of transferring charge to this distance ($r$). Since the probability of finding a neighbor grows as a function of the area in the 2D monolayer, we can write:

$$P_n(r) \propto k_1 \pi r^2 \approx k_2 r^2$$

And since we assume a tunneling process:

$$T(r) \propto k_3 \exp(-\beta r)$$

Therefore:

$$k_{AA} = k_2 r^2 \cdot k_3 \exp(-\beta r) = k_4 r^2 \cdot \exp(-\beta r),$$

where $\beta$ is the tunneling decay coefficient. For alkyl chains $\beta \approx 1[\text{Å}]^{-1}$ and $k_1$, $k_2$ , $k_3$, and $k_4$ are constants.
